# Mortality predictors in community-dwelling centenarians through comprehensive geriatric assessment

**DOI:** 10.1186/s12877-026-07459-x

**Published:** 2026-04-17

**Authors:** Zehra Sarikaya Demirbas, Mehmet Ilkin Naharci

**Affiliations:** Department of Geriatrics, Gulhane Faculty of Medicine & Gulhane Training and Research Hospital, Ankara, Turkey

**Keywords:** Centenarians, Community-dwelling older adults, Comprehensive geriatric assessment, Mortality predictors, Geriatric syndromes

## Abstract

**Background:**

There has been a steady rise in the global population of centenarians. However, information on the factors affecting survival in community-dwelling centenarians remains limited. Comprehensive geriatric assessment (CGA), with its multidisciplinary approach, offers important advantages in identifying predictors of mortality in older adults. This research sought to determine the most common chronic diseases and factors associated with mortality in community-dwelling centenarians using the CGA.

**Methods:**

The clinical documentation of community-dwelling individuals aged ≥ 100 years who attended a tertiary geriatric outpatient clinic between December 2016 and March 2025 were reviewed. Demographic characteristics, chronic diseases, geriatric syndromes, Charlson Comorbidity Index (CCI), anticholinergic cognitive burden (ACB) score, and laboratory findings at baseline were recorded. Mortality status was followed for 24 months via the National Death Registry and family reports. Multivariate logistic regression was employed to evaluate the independent factors linked to mortality.

**Results:**

The study population comprised 65 centenarians (mean age, 102.3 ± 3.3 years; 87.7% female). The two-year all-cause mortality rate was 69.2% (*n* = 45). The most common chronic diseases were hypertension (66.2%), chronic kidney disease (35.4%), and diabetes mellitus (16.9%). The most prevalent geriatric syndromes were urinary incontinence (60.0%), insomnia (33.8%), and dementia (26.2%). In multivariate analysis, insomnia (OR = 6.04; 95% CI: 1.16–31.4; *p* = 0.03) and pressure ulcers (OR = 11.9; 95% CI: 1.03–138.2; *p* = 0.04) were identified as independent predictors of mortality. Sensitivity analyses excluding individuals aged ≥ 110 years and those with hyperpolypharmacy confirmed these associations.

**Conclusions:**

In community-dwelling centenarians, insomnia and pressure ulcers are independent factors that significantly increase the risk of two-year mortality. These syndromes may reflect frailty and reduced physiological reserve in advanced age. Early diagnosis and targeted management provided by CGA may contribute to improved survival and quality of life in this vulnerable population.

## Introduction

There has been a marked increase in the number of people aged over 100 years worldwide. In 2000, there were approximately 151,000 centenarians, and by 2021, this number had risen to 573,000, representing an almost fourfold increase [1]. Projections suggest that the centenarian population could reach 3.5 million by 2050 [2]. These individuals represent a unique group that challenges the limits of human longevity and offers valuable insights into the aging process of the human body. In geriatrics, key areas of research include the health status of these individuals, the prevalence of age-related chronic diseases, and how these conditions influence mortality [3].

The prevalence of chronic conditions in this age group varies widely across studies [4, 5]. As an illustration, dementia has been reported in 27% to 89% of individuals, congestive heart failure in 27% to 60%, and diabetes in 1% to 12%. [6]. Nonetheless, some individuals appear to reach 100 years of age without experiencing significant health issues. In one study, approximately 23% of centenarians were found to have no major chronic diseases, 18% reported no physical disability, and more than half (55%) had preserved cognitive function at 100 years of age [7].

Identifying factors associated with mortality in very old populations may have implications for the prevention and early treatment of some specific diseases or conditions to increase survival. Emerging evidence from observational studies on centenarians has shown that several clinical factors, involving malnutrition, anemia, and lower concentrations of low-density lipoprotein cholesterol have been linked to mortality [8–11]. However, to date, no study has investigated the contribution of health problems to mortality using the comprehensive geriatric assessment (CGA), a multidisciplinary approach that integrates physical, cognitive, psychological, functional, and social domains in a holistic manner, unlike standard evaluations. Furthermore, studies focusing on centenarians are often methodologically heterogeneous, primarily due to differences in inclusion criteria, sampling strategies, and measurement instruments employed [12]. As a result, the applicability of these findings to the wider centenarian population is restricted, warranting a careful interpretation of the outcomes. Additional studies are needed to clarify the best predictors of adverse health outcomes in centenarians, who will constitute an important population group in the future.

Thus, this study aimed to identify the most common chronic diseases and examine the factors associated with mortality through CGA of community-dwelling centenarians, with the goal of providing novel insights into predictors of survival in this exceptional age group.

## Methods

### Study design and sample

**Fig. 1:**
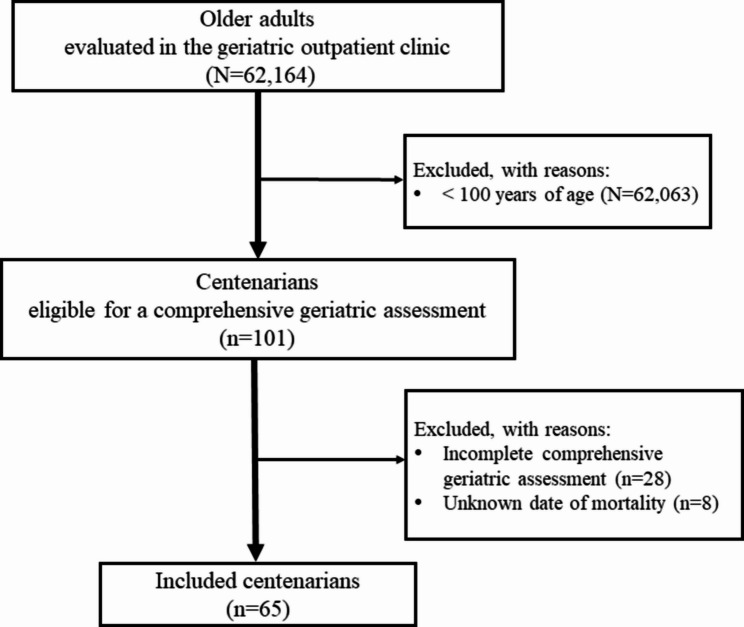
The flow chart of sample selection

Between December 2016 and March 2025, 62,164 older adults were evaluated at a tertiary-level geriatric outpatient clinic. Among them, community-dwelling centenarians (*n* = 101) were identified and assessed using their medical records and/or patient files. As illustrated in Fig. 1, individuals with unknown dates of death or incomplete comprehensive geriatric assessment were excluded. The research adhered to the ethical standards of the Declaration of Helsinki and received approval from the institutional review board (Gülhane Training and Research Hospital Ethics Committee; decision no: 2025/162).

The index date for each participant was the date of their first admission to the outpatient clinic. In the outpatient protocol, each participant underwent CGA by a geriatrician. The assessment at the index date included sociodemographic variables (age and gender), chronic diseases, geriatric syndromes, and laboratory parameters. In addition, clinical indicators such as anticholinergic burden (ACB), multimorbidity, and Charlson Comorbidity Index (CCI) were evaluated.

### Mortality status

The main endpoint of the study was all-cause mortality within the two-year follow-up. At the 24-month assessment, participants were categorized into two groups based on their survival status (yes or no).

Information on deaths was obtained from the Ministry of Health’s Death Registry and cross-verified with data provided by family members.

The prevalence of mortality among community-dwelling centenarians has been reported to be 42% [13]. Given this prevalence, the required minimum sample size was estimated as 38 participants, assuming a 95% confidence interval and a 5% margin of error.

### Chronic diseases

The chronic conditions considered included hypertension, chronic kidney disease (defined as eGFR < 60 mL/min/1.73 m^2^), diabetes mellitus, cardiovascular disease, chronic obstructive pulmonary disease, cerebrovascular disease, hypothyroidism, and Parkinson’s disease. Information on these conditions was obtained through patient or caregiver reports, verification of prescribed medications, and review of clinical documentation.

### Geriatric syndromes

We evaluated geriatric syndromes, including urinary incontinence, insomnia, dementia, malnutrition, pressure ulcers, and polypharmacy.

Urinary incontinence was assessed using binary (yes/no) responses to questions regarding unintentional urine leakage. Sleep-related disturbances were identified based on four specific criteria: difficulty in initiating sleep at night, difficulty in returning to sleep after nighttime awakenings, waking up too early in the morning, and the need to nap during the day. Participants who reported at least one of these complaints were categorized as having a sleep disorder [14]. Dementia was identified through a consensus of two experienced geriatricians, applying the diagnostic criteria outlined in both DSM-IV and DSM-V [15, 16]. Nutritional status was evaluated using the Mini Nutritional Assessment–Short Form (MNA-SF), which provides scores from 0 to 14; a score of ≤ 7 indicated malnutrition [17]. In addition, individuals receiving oral nutritional supplements were also categorized as malnourished. The detection of pressure ulcers followed the recommendations of the Prevention and Treatment of Pressure Ulcers/Injuries: Clinical Practice Guideline [18]. Polypharmacy was defined as the concurrent use of five or more medications, while the use of ten or more was considered hyperpolypharmacy [19].

### Other variables

Anticholinergic burden was assessed with the Anticholinergic Cognitive Burden Scale [20]. For every participant, a cumulative ACB score was obtained by adding up the anticholinergic ratings of all prescribed medications.Participants were divided into two categories: ACB score of zero (no) and ACB score ≥ 1 (yes).

Multimorbidity referred to the presence of two or more chronic illnesses occurring simultaneously in a person [21].

The Charlson Comorbidity Index was used to quantitatively assess the cumulative impact of chronic diseases. Conditions at the time of outpatient admission were categorized according to the 19 diagnostic groups included in the CCI scoring system. The sum of the points assigned to these diagnostic categories was recorded as the CCI score [22, 23]. Based on this score, participants were stratified into three groups: low-to-moderate comorbidity (0–2), high comorbidity (3–4), and very high comorbidity (≥ 5) [24, 25].

Anemia was characterized by hemoglobin concentrations below 13.0 g/dL in men and below 12.0 g/dL in women [26]. Vitamin B12 deficiency was considered present when serum B12 levels were below 200 pg/mL [27]. Additionally, glucose, HbA1c, and LDL levels were measured.

### Statistical analysis

Statistical analyses were conducted using the Statistical Package for Social Sciences (SPSS) version 29.0 (IBM SPSS Inc.). Data were summarized as counts and percentages, means with standard deviations, or medians, depending on the type and distribution of the variables. Group comparisons for categorical variables were conducted using the chi-square (χ^2^) test, and those with expected cell counts < 5 were reanalyzed using Fisher’s exact test. The distribution of continuous variables was evaluated using the Kolmogorov–Smirnov test, and the Mann–Whitney U test was employed when the assumption of normality was not met. Logistic regression analysis was performed to explore the relationship between mortality (dependent variable) and chronic diseases, geriatric syndromes, as well as relevant laboratory parameters (independent variables). Furthermore, binary logistic regression models were applied to identify predictors of increased mortality risk, including variables with *p* < 0.10 in the univariate analysis or those considered clinically relevant. The associations were reported as odds ratios (OR) with corresponding 95% confidence intervals (CI). Statistical significance was set at a *p*-value < 0.05. Model calibration was assessed with the Hosmer–Lemeshow test, and overall model fit was evaluated using the Omnibus test. All results were interpreted within a 95% confidence interval framework.

## Results

### Baseline characteristics

A total of 65 community-dwelling centenarians were enrolled in the study, with a mean age of 102.3 ± 3.3 years. After two years of follow-up, 45 individuals (69.2%) had passed away, whereas 20 (30.8%) remained alive. Most participants were aged 100–104 years (78.5%) and the majority were women (87.7%).Among chronic diseases, hypertension was the most prevalent (66.2%), followed by chronic kidney disease (35.4%) and diabetes mellitus (16.9%). The most frequently observed geriatric syndromes were urinary incontinence was (60.0%), insomnia (33.8%), and dementia (26.2%). In addition, the majority had a very high comorbidity burden (CCI ≥ 5). Table 1 presents the baseline demographic and clinical characteristics of the participants.

**Table 1:** Characteristics of the study group

**Variables**	**Total (n=65)**	**Two years of mortality**			***p***
		**Yes (*n* = 45)**	**No (*n* = 20)**		
Age (years)					
100–104	51 (78.5)	37 (82.2)		14 (70.0)	0.286
105–109	11 (16.9)	7 (15.6)		4 (20.0)	
110 +	3 (4.6)	1 (2.2)		2 (10.0)	
Gender (female), n (%)	57 (87.7)	38 (84.4)		19 (95.0)	0.232
*Chronic diseases*					
Hypertension, n (%)	43 (66.2)	27 (60.0)		16 (80)	0.116
Chronic kidney disease, n (%)	23 (35.4)	14 (31.1)		9 (45.0)	0.239
Diabetes mellitus, n (%)	11 (16.9)	5 (11.1)		6 (30.0)	0.061
Cardiovascular disease, n (%)	9 (13.8)	7 (15.6)		2 (10.0)	0.710
COPD, n (%)	8 (12.3)	6 (13.3)		2 (10.0)	0.529
Cerebrovascular disease, n (%)	5 (7.7)	3 (6.7)		2 (10.0)	0.639
Hypothyroidism n (%)	5 (7.7)	2 (4.4)		3 (15.0)	0.165
Parkinson's disease n (%)	2 (3.1)	1 (2.2)		1 (5.0)	0.524
*Geriatric syndromes*					
Urinary incontinence, n (%)	39 (60.0)	28 (62.2)		11 (55.0)	0.583
Insomnia, n (%)	22 (33.8)	19 (42.2)		3 (15.0)	**0.046**
Dementia, n (%)	17 (26.2)	13 (28.9)		4 (20.0)	0.551
Malnutrition, n (%)	15 (23.1)	10 (22.2)		5 (25.0)	0.806
Pressure ulcer, n (%)	13 (20.0)	12 (26.7)		1 (5.0)	**0.048**
Polypharmacy, n (%)					
No	50 (76.9)	35 (77.8)		15 (75.0)	0.831
Yes	14 (21.5)	9 (20.0)		5 (25.0)	
Hyper-	1 (1.5)	1 (2.2)		0 (0.0)	
Anticholinergic burden (yes), n (%)	21 (32.3)	17 (37.8)		4 (20.0)	0.250
Multimorbidity, n (%)	30 (60.0)	25 (55.6)		14 (70.0)	0.273
Charlson comorbidity index, n (%)					
Low to medium	0 (0.0)	0 (0.0)		0 (0.0)	0.383
High	20 (30.8)	12 (26.7)		8 (40.0)	
Very high	45 (69.2)	33 (73.3)		12 (60.0)	
*Laboratory assesments*					
Anemia, n (%)	32 (49.2)	26 (57.8)		6 (30.0)	**0.045**
Vitamin B12 deficiency, n (%)	25 (38.5)	17 (37.8)		8 (40.0)	0.776
Glucose (mg/dL), (median)	107.26	105.07		112.2	0.887
HbA1c (%), (median)	6.3	5.7		6.9	**0.047**
LDL cholesterol (mg/dL), (median)	103.8	99.2		111.4	0.325

*Abbreviations*: *COPD* Chronic Obstructive Pulmonary Disease, *ONS* Oral Nutritional Supplements, *LDL* Low-Density Lipoprotein

Values given in bold indicate sitatistically significant results (*p* < 0.05)

### Mortality-associated factors

**Fig. 2:**
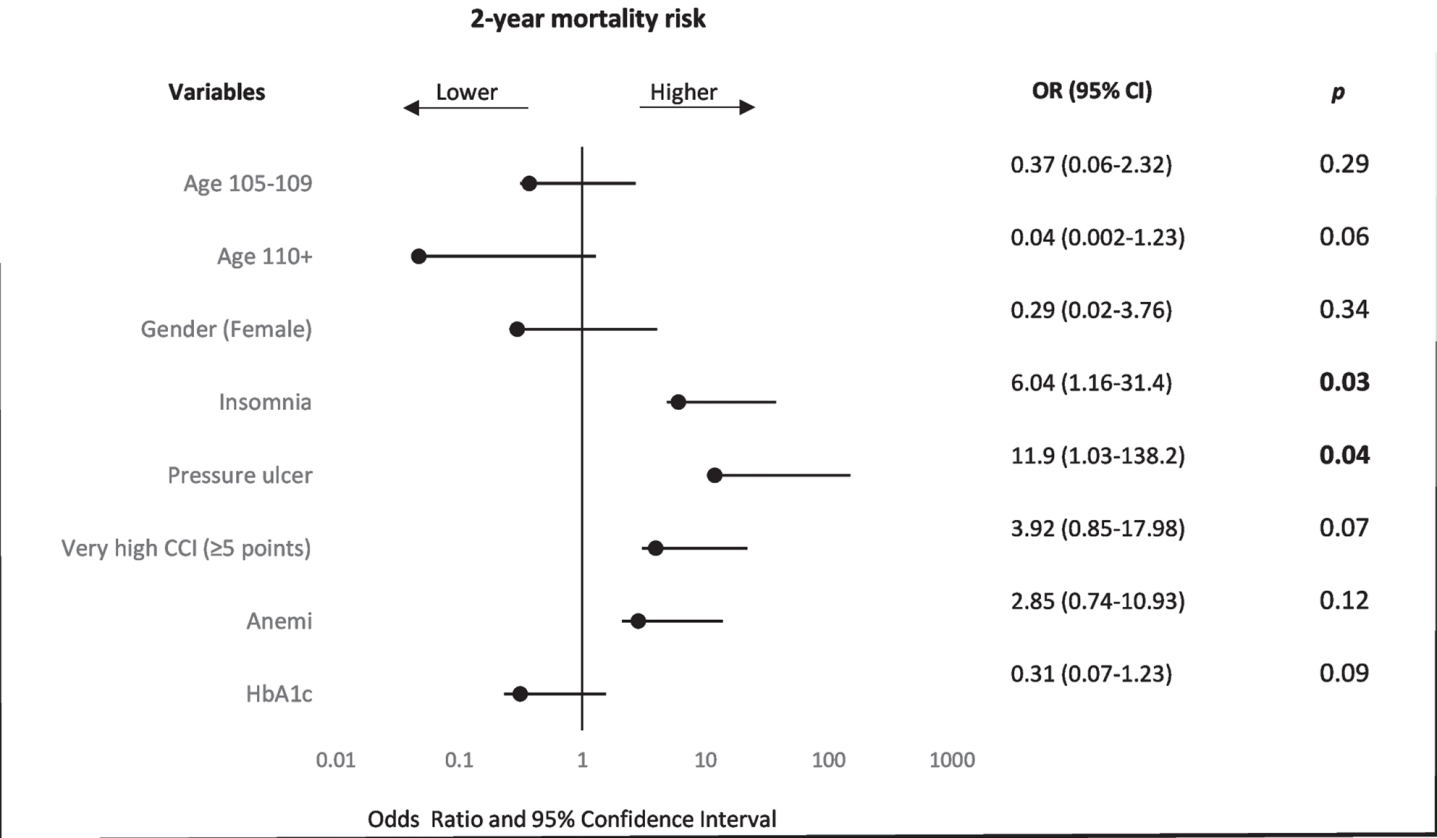
Forest plot of predictors of two-year mortality with corresponding odds ratios (95% CI) and *p* values

In the multivariate logistic regression analysis, after adjustment for potential confounders, insomnia (OR: 6.04; 95% CI: 1.16–31.4; *p* = 0.03) and pressure ulcers (OR: 11.9; 95% CI: 1.03–138.2; *p* = 0.04) emerged as independent predictors significantly linked to a higher risk of two-year mortality.Gender, anemia, a very high Charlson Comorbidity Index (CCI ≥ 5) and age between 105–109 years were not found to be significantly associated with mortality (Fig. 2).

The inferential goodness-of-fit test, Hosmer–Lemeshow (HL) test, produced a chi-square value of 0.062 and was found to be non-significant (*p* = 0.969), indicating that the model fits the data well. On the other hand, the Omnibus test confirmed that the model was highly significant (− 2LL = 67.555, χ2(2) = 11.944, p: < 0.001).

### Sensitivity analysis

Sensitivity analysis did not alter the results. Participants aged ≥ 110 years and those with hyperpolypharmacy were excluded due to the small number of cases and their potential to introduce bias related to extreme age or medication burden. After these exclusions, the association between two-year all-cause mortality and both insomnia (OR = 7.71; 95% CI: 1.51–39.38; *p* = 0.014) and pressure ulcers (OR = 9.47; 95% CI: 1.08–82.89; *p* = 0.042) remained statistically significant, even after adjusting for confounding variables.

## Discussion

To the best of our knowledge, this is the first comprehensive investigation to examine the associations of geriatric syndromes, chronic diseases, disease burden, and laboratory variables with mortality among community-dwelling individuals in this exceptional age group. This study offers significant insights into the health status and survival outcomes of centenarians. Our analysis revealed that insomnia and pressure ulcers remained significantly associated with a higher risk of two-year all-cause mortality, even after controlling for confounding variables. These results highlight the necessity of identifying mortality-related risk factors and developing targeted strategies such as early screening for insomnia, prevention and timely management of pressure ulcers, and optimization of sleep hygiene to promote healthy aging among the oldest-old. Moreover, the two-year all-cause mortality rate in this cohort was 69.2%, underlining the critical importance of continuous medical surveillance and care in this highly vulnerable group.

Mortality rates are important indicators of overall health and functional capacity in centenarians. The annual mortality rates suggest that survival in this age group is highly variable and closely related to individual health characteristics. For instance, the 100-plus Study conducted in the Netherlands found that cognitively intact centenarians had an annual mortality rate of 21% and a two-year rate of 28%, while mortality rose to 42% among those with cognitive impairment [13]. In our study, the two-year mortality rate was higher, which may be attributed to the older mean age andhigher prevalence of dementia in our cohort. Furthermore, US-based cohort study of male centenarians reported an annual mortality rate of 32%, with variations based on marital status and general health [28]. In the longitudinal phase of the Georgia Centenarian Study, the annual mortality rate was estimated to be approximately 50%, emphasizing the methodological challenges of conducting long-term follow-up studies in this population [29]. Moreover, a recent study of centenarians who sustained hip fractures reported mortality rates of 19.6%, 26.8%, and 39.3% at 90 days, 6 months, 1 year, respectively [30]. These findings suggest that mortality in centenarians is strongly influenced by factors such as cognitive function, physical capacity, comorbidity burden, and mobility, highlighting the heterogeneity of survival trajectories in this age group.

Insomnia, defined as difficulty initiating or maintaining sleep accompanied by nonrestorative rest, is a highly prevalent geriatric syndrome. Insomnia has been shown to increase proinflammatory cytokine levels and disrupt glucose and lipid metabolism, potentially accelerating frailty and vascular aging processes in centenarians. In our study, insomnia, identified by the presence of at least one of four specific complaints, was significantly associated with two-year mortality among community-dwelling centenarians. This association appears stronger than that reported in previous studies, such as the Hainan Centenarian Cohort, which assessed only daytime sleep duration and found an increased mortality risk among male centenarians with ≥ 2 h of daytime sleep [31, 32], and the Rome Centenarian Study, which focused on overall sleep quality [33]. While our findings are consistent with the existing literature, the present study is distinct in that it evaluatedclinically defined insomnia rather than total sleep duration or subjective sleep quality. Moreover, the persistence of this association after adjustment for other key clinical variables suggests that insomnia may be a robust and clinically meaningful prognostic indicator in community-dwelling centenarians. Collectively, these findings support the notion that insomnia in this population may reflect underlying circadian or physiological dysregulation, contributing to an elevated risk of mortality.

Pressure ulcers are commonly associated with frailty, immobility, and general health deterioration in older adults. Pressure ulcers may precipitate systemic inflammation, protein-energy malnutrition, and prolonged immobility, thereby accelerating frailty and increasing mortality risk in centenarians [34, 35]. Current evidence suggests a link between pressure ulcers and increased mortality [36], however; to date, no study has specifically investigated this relationship in centenarians. In our analysis, pressure ulcers emerged as strong and independent predictor of two-year all-cause mortality; individuals with pressure ulcers had an approximately 12-fold higher risk of death. This association suggests that, in the oldest-old, pressure ulcers may not merely represent localized tissue damage but rather serve as a sentinel marker of profound physiological decline, reduced resilience, and impending mortality. Our findings address the prognostic significance of pressure ulcers as a potentially modifiable target for the comprehensive care of centenarians.

This research is subject to some limitations. Its single-center design and the relatively small cohort size may restrict the extent to which the results can be generalized. However, the inclusion of all eligible centenarians with long-term follow-up will be useful for research that aiming to achieve a healthy life. The restriction to community-dwelling individuals, with the exclusion of those in institutional care, may have introduced selection bias; nevertheless, it allowed for the examination of a more homogeneous group with similar living conditions and ensured a standardized data collection. Furthermore, the assessment of insomnia was based on self-reported data, which may represent a potential source of measurement bias. This limitation has been carefully considered in the interpretation of the findings. Importantly, the exclusive focus on community-dwelling centenarians represents a notable strength of this study, as it enables a clearer identification of the specific challenges that may contribute to mortality risk. Another limitation of this study is the lack of detailed sociodemographic data, including education, occupation, income, and family structure. These factors are important social determinants that may influence health and longevity in older adults. However, such information was not systematically captured in the electronic medical records of this centenarian cohort. Future multicenter studies incorporating standardized social and socioeconomic measures are needed to better understand their contribution to longevity and mortality in the oldest-old population. Although standardized measures of activities of daily living (ADLs) and instrumental activities of daily living (IADLs) were not available in our dataset, functional capacity was partly reflected by cognitive and nutritional status and by the presence of geriatric syndromes such as pressure ulcers. Future studies including validated ADL and IADL assessments may provide deeper insight into functional heterogeneity and survival among centenarians.

In summary, this research offers valuable insights into the clinical features and mortality-associated factors of community-dwelling centenarians. Our findings demonstrate that geriatric syndromes, such as pressure ulcers and insomnia, alongside chronic diseases, are highly prevalent even at extreme ages and are significantly associated with an increased mortality risk. These results emphasize the need for continued clinical attention to modifiable risk factors in this unique and growing population. With the worldwide increase in people living to 100 years and older, gaining deeper knowledge of the determinants of survival in this population is crucial for shaping individualized and holistic geriatric care. Beyond its prognostic significance, insomnia should be regarded as a potentially modifiable risk factor in very old adults. Although often underrecognized in clinical practice, sleep disturbances can be improved through both behavioral and pharmacological interventions, even among centenarians. Nonpharmacological strategies, such as optimizing sleep hygiene, addressing pain, nocturia, or mood disorders, and encouraging regular daytime activity, may enhance sleep quality and overall well-being. In this age group, identifying and managing insomnia may represent one of the few realistic opportunities to promote prevention and improve the quality of life. Future studies focusing on targeted interventions for sleep disorders in centenarians are warranted to determine whether such approaches can translate into better survival and functional outcomes. It may serve as a foundation for developing predictive models and personalized intervention strategies aimed at extending both lifespan and healthspan in centenarians. To validate and expand these findings, future prospective, large-scale, multicenter studies are warranted.

## Data Availability

The datasets generated and analyzed during the current study are available from the corresponding author on reasonable request.

## References

[1] Dai Z, Lee SY, Sharma S, Ullah S, Tan ECK, Brodaty H, et al. A systematic review of diet and medication use among centenarians and near-centenarians worldwide. Geroscience. 2024;46(6):6625–39. 10.1007/s11357-024-01247-4. 38967696 10.1007/s11357-024-01247-4 PMC11493889

[2] Stepler R. World’s centenarian population projected to grow eightfold by 2050. Pew Research Center; 2016. Available from: https://www.pewresearch.org/short-reads/2016/04/21/worlds-centenarian-population-projected-to-grow-eightfold-by-2050. Accessed 15 Jan 2024.

[3] Espinosa O, Bejarano V, Franky I, Pagali S, Drummond M, Franco OH. Mortality causes and health spending by gender and health conditions in octogenarians, nonagenarians and centenarians in Colombia. Sci Rep. 2025;15(1):918. 10.1038/s41598-024-84150-4. 39762274 10.1038/s41598-024-84150-4 PMC11704332

[4] Jopp DS, Boerner K, Rott C. Health and disease at age 100. Dtsch Arztebl Int. 2016;113(12):203–10. 10.3238/arztebl.2016.0203. 27118718 10.3238/arztebl.2016.0203 PMC5400033

[5] Gimeno-Miguel A, Clerencia-Sierra M, Ioakeim I, Poblador-Plou B, Aza-Pascual-Salcedo M, González-Rubio F, et al. Health of Spanish centenarians: a cross-sectional study based on electronic health records. BMC Geriatr. 2019;19(1):226. 10.1186/s12877-019-1235-7. 31426764 10.1186/s12877-019-1235-7 PMC6701024

[6] Hazra NC, Dregan A, Jackson S, Gulliford MC. Differences in health at age 100 according to sex: population-based cohort study of centenarians using electronic health records. J Am Geriatr Soc. 2015;63(7):1331–7. 10.1111/jgs.13484. 26096699 10.1111/jgs.13484 PMC4745036

[7] Ailshire JA, Beltrán-Sánchez H, Crimmins EM. Becoming centenarians: disease and functioning trajectories of older US adults as they survive to 100. J Gerontol A Biol Sci Med Sci. 2015;70(2):193–201. 10.1093/gerona/glu124. 25136001 10.1093/gerona/glu124 PMC4311187

[8] Wang H, Hai S, Liu Y, Liu Y, Dong B. Skeletal muscle mass as a mortality predictor among nonagenarians and centenarians: a prospective cohort study. Sci Rep. 2019;9(1):2420. 10.1038/s41598-019-38893-0. 30787413 10.1038/s41598-019-38893-0 PMC6382937

[9] Jia W, Wang S, Yang S, Zhao Y, Zhu Q, Ning C, et al. Association of anemia with all-cause mortality in Chinese centenarians: a prospective cohort study. J Nutr Health Aging. 2024;28(7):100248. 10.1016/j.jnha.2024.100248. 38669839 10.1016/j.jnha.2024.100248 PMC12433784

[10] Liu S, Cao W, Li Z, Wang S, Yang S, Lu M, et al. Association between different adiposity measures and all-cause mortality risk among centenarians: a prospective cohort study. Clin Nutr. 2023;42(7):1219–26. 10.1016/j.clnu.2023.04.023. 37236872 10.1016/j.clnu.2023.04.023

[11] Zou X, Li JH, Hu YX, Wang HJ, Sun SS, Xu WH, et al. Serum lipid profiles and all-cause mortality: a retrospective single center study on Chinese inpatient centenarians. Front Public Health. 2022;10:776814. 10.3389/fpubh.2022.776814. 35646784 10.3389/fpubh.2022.776814 PMC9136240

[12] Cheng A, Leung Y, Harrison F, Brodaty H. The prevalence and predictors of anxiety and depression in near-centenarians and centenarians: a systematic review. Int Psychogeriatr. 2019;31(11):1539–48. 10.1017/S1041610219000802. 31354113 10.1017/S1041610219000802

[13] Holstege H, Beker N, Dijkstra T, Pieterse K, Wemmenhove E, Schouten K, et al. The 100-plus study of cognitively healthy centenarians: rationale, design and cohort description. Eur J Epidemiol. 2018;33(12):1229–49. 10.1007/s10654-018-0451-3. 30362018 10.1007/s10654-018-0451-3 PMC6290855

[14] Naharci MI, Bozoglu E, Kocak N, et al. Analysis of the effects of galantamine and donepezil on sleep disturbances in patients with dementia. Bull Clin Psychopharmacol. 2011;21(4):339–44.

[15] American Psychiatric Association. Diagnostic and statistical manual of mental disorders. 4th ed. Washington, DC: American Psychiatric Association; 1994.

[16] American Psychiatric Association. Diagnostic and statistical manual of mental disorders. 5th ed. Washington, DC: American Psychiatric Association; 2013.

[17] Kaiser MJ, Bauer JM, Ramsch C, et al. Validation of the mini nutritional assessment short-form (MNA-SF): a practical tool for identification of nutritional status. J Nutr Health Aging. 2009;13(9):782–8. 19812868 10.1007/s12603-009-0214-7 PMC12878690

[18] Wound/Pressure Ulcer/Burn Guidelines Drafting Committee, Fujiwara H, Irisawa R, Otsuka M, et al. Wound, pressure ulcer, and burn guidelines (2023)-2: guidelines for the diagnosis and treatment of pressure ulcers, third edition. J Dermatol. 2025. 10.1111/1346-8138.17758. 10.1111/1346-8138.17758 40569688

[19] Masnoon N, Shakib S, Kalisch-Ellett L, Caughey GE. What is polypharmacy? A systematic review of definitions. BMC Geriatr. 2017;17(1):230. 10.1186/s12877-017-0621-2. 29017448 10.1186/s12877-017-0621-2 PMC5635569

[20] Boustani M, Campbell N, Munger S, Maidment I, Fox C. Impact of anticholinergics on the aging brain: a review and practical application. Aging Health. 2008;4(3):311–20. 10.2217/1745509X.4.3.311.

[21] Skou ST, Mair FS, Fortin M, Guthrie B, Nunes BP, et al. Multimorbidity. Nat Rev Dis Primers. 2022;8(1):48. 10.1038/s41572-022-00376-4. 35835758 10.1038/s41572-022-00376-4 PMC7613517

[22] Piñeiro-Fernández JC, et al. Comorbidity burden, management, and in-hospital outcomes in centenarians with proximal hip fracture: a nationwide cohort study (2004-2020). Arch Osteoporos. 2025;20(1):88. 10.1007/s11657-025-01576-7. 40646291 10.1007/s11657-025-01576-7 PMC12254154

[23] Roedl K, Daniels R, Theile P, Kluge S, Müller J, Behrendt CA. The independent impact of peripheral arterial disease on mortality in nonagenarians and centenarians who were treated in an intensive care unit: a consecutive cohort of 1 108 patients. Eur J Vasc Endovasc Surg. 2023;65(4):582–9. 10.1016/j.ejvs.2023.01.026. 36682405 10.1016/j.ejvs.2023.01.026

[24] Charlson ME, Pompei P, Ales KL, MacKenzie CR. A new method of classifying prognostic comorbidity in longitudinal studies: development and validation. J Chronic Dis. 1987;40(5):373–83. 10.1016/0021-9681(87)90171-8. 3558716 10.1016/0021-9681(87)90171-8

[25] Charlson M, Szatrowski TP, Peterson J, Gold J. Validation of a combined comorbidity index. J Clin Epidemiol. 1994;47(11):1245–51. 10.1016/0895-4356(94)90129-5. 7722560 10.1016/0895-4356(94)90129-5

[26] Cappellini MD, Motta I. Anemia in clinical practice—definition and classification: does hemoglobin change with aging? Semin Hematol. 2015;52(4):261–9. 10.1053/j.seminhematol.2015.07.006. 26404438 10.1053/j.seminhematol.2015.07.006

[27] Allen LH. How common is vitamin B-12 deficiency? Am J Clin Nutr. 2009;89(2):693S-S696. 10.3945/ajcn.2008.26947A. 19116323 10.3945/ajcn.2008.26947A

[28] Quach LT, Cho K, Driver JA, Ward R, Spiro A, Dugan E, et al. Social characteristics, health, and mortality among male centenarians using Veterans Affairs health care. Res Aging. 2022;44(2):136–43. 10.1177/01640275211000724. 33779393 10.1177/01640275211000724 PMC10756333

[29] Poon LW, Jazwinski M, Green RC, Woodard JL, Martin P, Rodgers WL, et al. Methodological considerations in studying centenarians: lessons learned from the Georgia Centenarian Studies. Annu Rev Gerontol Geriatr. 2007;27(1):231–64. 21852888 PMC3156654

[30] Jang BW, Kim JW, Nho JH, Lee YK, Park JW, Cha YH, et al. Hip fractures in centenarians: functional outcomes, mortality, and risk factors from a multicenter cohort study. Clin Orthop Surg. 2023;15(6):910–6. 10.4055/cios23223. 38045583 10.4055/cios23223 PMC10689221

[31] Qiu L, Sautter J, Liu Y, Gu D. Age and gender differences in linkages of sleep with subsequent mortality and health among very old Chinese. Sleep Med. 2011;12(10):1008–17. 10.1016/j.sleep.2011.04.014. 22036598 10.1016/j.sleep.2011.04.014 PMC3685295

[32] Yang S, Li R, Liu G, Wang S, Li X, Chen S, et al. Sleep status of centenarians and its association with death in the China Hainan Centenarian Cohort Study. Sleep Health. 2024;10(6):713–21. 10.1016/j.sleh.2024.08.002. 39341740 10.1016/j.sleh.2024.08.002

[33] Tafaro L, Cicconetti P, Baratta A, Brukner N, Ettorre E, Marigliano V, et al. Sleep quality of centenarians: cognitive and survival implications. Arch Gerontol Geriatr. 2007;44(1):385–9. 10.1016/j.archger.2007.01.054. 17317480 10.1016/j.archger.2007.01.054

[34] Cornish L. Prevention of pressure ulcers in older people with frailty. Nurs Older People. 2022. 10.7748/nop.2022.e1405 10.7748/nop.2022.e1405 35861045

[35] Landi F, Onder G, Russo A, Bernabei R. Pressure ulcer and mortality in frail elderly people living in community. Arch Gerontol Geriatr. 2007;44(1):217–23. 10.1016/j.archger.2007.01.030. 17317456 10.1016/j.archger.2007.01.030

[36] Lan X, Tang Y, Huang Z, Zhou T, Wang C, Ma Y, et al. Global, regional, and national burden of pressure ulcers from 1990 to 2021 and projections over the next decade: results from the 2021 GBD study. Wound Repair Regen. 2025;33(4):e70064. 10.1111/wrr.70064. 40642880 10.1111/wrr.70064 PMC12247018

